# Mechanisms of iron metabolism in *Caenorhabditis elegans*

**DOI:** 10.3389/fphar.2014.00113

**Published:** 2014-05-21

**Authors:** Cole P. Anderson, Elizabeth A. Leibold

**Affiliations:** Department of Medicine, Division of Hematology and Hematologic Malignancies and Department of Oncological Sciences, University of Utah, Salt Lake CityUT, USA

**Keywords:** ferritin, DMT1, SMF-3, iron deficiency, hypoxia, hypoxia-inducible factor, insulin signaling, *C. elegans*

## Abstract

Iron is involved in many biological processes essential for sustaining life. In excess, iron is toxic due to its ability to catalyze the formation of free radicals that damage macromolecules. Organisms have developed specialized mechanisms to tightly regulate iron uptake, storage and efflux. Over the past decades, vertebrate model organisms have led to the identification of key genes and pathways that regulate systemic and cellular iron metabolism. This review provides an overview of iron metabolism in the roundworm *Caenorhabditis elegans* and highlights recent studies on the role of hypoxia and insulin signaling in the regulation of iron metabolism. Given that iron, hypoxia and insulin signaling pathways are evolutionarily conserved, *C. elegans* provides a genetic model organism that promises to provide new insights into mechanisms regulating mammalian iron metabolism.

## INTRODUCTION

Iron is essential due to its presence in proteins involved in key metabolic processes such as DNA synthesis, mitochondrial respiration, and oxygen transport. Regulation of cellular iron content is crucial as excess iron catalyzes the generation of reactive oxygen species that damage DNA and proteins, while cellular iron deficiency causes cell cycle arrest and cell death. Disruption of iron metabolism, by iron excess or iron deficiency, leads to common hematological, neurodegenerative, and metabolic diseases (Fleming and Ponka, 2012). As a consequence, organisms have developed strategies to sense, transport and store this metal.

Our understanding of the mechanisms that regulate iron metabolism has advanced through the use of model organisms. Physiological and genetic studies in transgenic mice have revealed the mechanism regulating systemic iron metabolism by the ferroportin–hepcidin axis. *Saccharomyces cerevisiae* have been used to unravel the complex pathways involved in Fe-S cluster synthesis (Lill and Muhlenhoff, 2008), while zebrafish have been critical in the identification of genes involved in hematopoiesis (Shafizadeh and Paw, 2004). More recently, the soil nematode *Caenorhabditis elegans* has emerged as a model of iron metabolism. The advantages of *C. elegans* include a short generation time and life span, the feasibility of genetic screens and the opportunity to study physiological processes in a whole organism context*. C. elegans* orthologs have been identified for many human genes (Shaye and Greenwald, 2011) and many of the key genes and pathways regulating mammalian iron metabolism are conserved in *C. elegans.* The genetic tractability of *C. elegans* can provide a complementary approach to mammalian systems to identify novel genes and unravel complex pathways involved in iron metabolism. This review provides an overview of our current understanding of iron metabolism in *C. elegans*, how iron metabolism integrates with oxygen and insulin signaling, and how this genetic model can provide insights in mammalian iron metabolism.

## CONSERVATION OF IRON METABOLISM IN *C. elegans*

**FIGURE 1:**
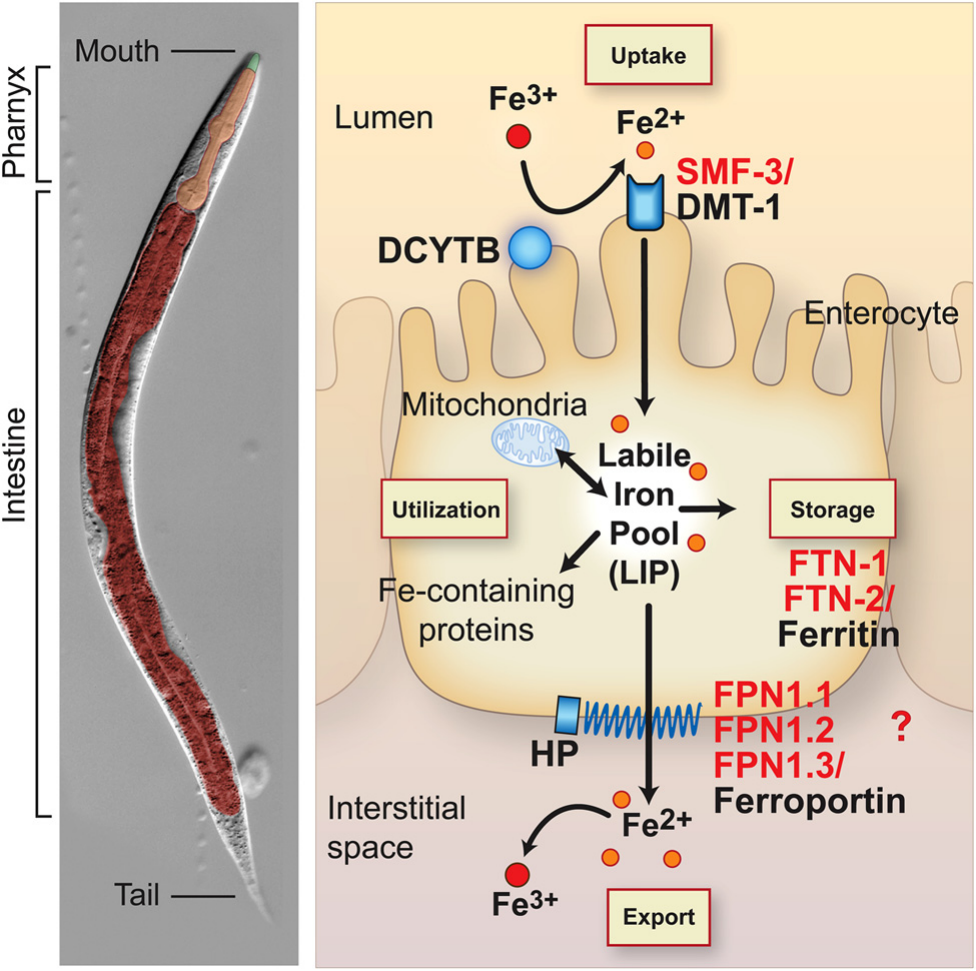
**Conservation of intestinal iron metabolism in mammals and *Caenorhabditis elegans*.** *C. elegans* anatomy is shown in the *left panel*. The body plan of *C. elegans* is made up of two concentric tubes separated by the interstitial space (pseudocoelum). The inner tube consists of the intestine and the outer tube consists of cuticle, hypodermis, muscle and nervous tissue. The digestive tract is an epithelial tube containing the mouth, pharynx (foregut) and intestine (midgut). *Right panel*, an intestinal epithelial cell is shown with an apical brush border membrane facing the lumen and a basolateral membrane facing the interstitial space or blood in mammals. Mammalian proteins (black) and *C. elegans* orthologs (red) are indicated. Dietary non-heme iron is reduced by ferrireductases (e.g. DCYTB1) and transported across the apical intestinal membrane by SMF-3/DMT1. Cytosolic iron is incorporated in iron-containing proteins and transported to mitochondria for Fe-S cluster biosynthesis and heme biosynthesis in mammals. *C. elegans* are heme auxotrophs and are dependent on acquiring heme from the environment (Rao et al., 2005). Iron is also exported across the basolateral membrane into the interstitial space (blood in mammals) by FPN-1.1, FPN-1.2, FPN-1.3/ferroportin. Ferroportin is the sole iron exporter in mammals, whereas *C. elegans* express three orthologs whose specific functions in iron export are not well understood. Iron export by ferroportin is coupled to the oxidation of iron by the multicopper oxidase hephaestin (HEPH). The *C. elegans* genome harbors putative *DCYTB* and *HEPH* homologs. Iron not utilized or exported is stored in FTN-1, FTN-2/ferritin. Iron deficiency stabilizes *C. elegans* HIF-1 and mammalian HIF-2α, leading to the transcriptional activation of *smf-3/DMT1* to increase iron absorption. Mammalian DCYTB and ferroportin are also activated by HIF-2α during iron deficiency. HIF-1 regulation of *C. elegans* DCYTB and FPN-1-1, FPN1.2, and FPN-1.3 orthologs remains to be determined. Iron deficiency reduces ferritin abundance in *C. elegans* and mammals by different mechanisms: *C. elegans* lack the IRP-IRE network and ferritin is transcriptionally repressed by HIF-1, whereas mammalian ferritin is translationally repressed by IRPs.

All organisms must maintain cellular iron content within a narrow range to avoid the adverse consequences of iron depletion or excess. This is accomplished in vertebrates by precise mechanisms that regulate iron uptake, storage and efflux (Andrews and Schmidt, 2007; Zhang and Enns, 2009; **Figure 1**). Mammals acquire iron solely from the diet. Dietary non-heme iron is reduced by membrane bound ferrireductases (e.g. DCYTB, also known as CYBRD) and transported across the apical membrane of intestinal enterocytes by divalent-metal transporter 1 (DMT1, also known as NRAMP2, SLC11A2 and DCT1; Mackenzie and Garrick, 2005; Shawki et al., 2012). Iron is released into a cellular labile iron pool thought to consist of low molecular weight iron complexes. This pool is kept small due to the ability of iron to catalyze the production of reactive oxygen species. Iron is utilized by the mitochondria for Fe-S cluster and heme biosynthesis, and by iron-containing proteins in the cytosol and nucleus. Iron is exported across the basolateral membrane into the circulation by ferroportin (FPN1, also known as SLC40A1, IREG1 and MTP1) in concert with its oxidation by the multicopper oxidase hephaestin (HEPH). Iron enters the circulation where it binds with high affinity to transferrin for delivery to cells expressing transferrin receptor 1 (TfR1, also known as TFRC). TfR1-transferrin-Fe(III) complexes are internalized by receptor mediated endocytosis. Iron is released from transferrin, reduced to Fe(II) by the ferrireductase STEAP3 and transported across the endosomal membrane to the cytoplasm by DMT1. Thus, DMT1 is essential in intestinal non-heme iron absorption as well as transport of endosomal iron released by transferrin into the cytoplasm. Although most cell types express TfR1, erythroid precursors are dependent on Tf-TfR1-DMT1 for iron uptake as disruption of *Tfrc* gene in mice (Levy et al., 1999) or mice with reduced transferrin (Trenor et al., 2000) developed severe anemia. DMT1 mutations in humans (Shawki et al., 2012), the *mk* mouse (Fleming et al., 1997), and the Belgrade rat (Fleming et al., 1998) also cause a severe microcytic hypochromic anemia, underscoring the importance of DMT1 in intestinal and erythroid iron acquisition.

Mammals can also acquire iron by the intestinal absorption of heme iron that comes primarily from animal sources. Although several heme importers have been identified (Yuan et al., 2013), the mechanism regulating intestinal heme import is not well understood. It is likely that heme oxygenase 1 releases iron from dietary heme, which is then exported by ferroportin into the circulation.

When body iron stores are high, cytosolic iron is not exported, and is instead sequestered in ferritin in an inert form unable to catalyze free radical formation (Harrison and Arosio, 1996; Torti and Torti, 2002; Theil, 2011). After 3 days, iron in ferritin is lost by enterocyte sloughing into the intestinal lumen. The regulation of intestinal ferritin is crucial as it serves as a cellular iron “sink” to limit efflux of iron into the circulation (Vanoaica et al., 2010; Galy et al., 2013). Because there is no regulated mechanism for iron excretion, precise regulation of intestinal iron uptake and storage is required. Given the fundamental nature of iron metabolism, it is not surprising that many proteins involved in intestinal iron uptake, storage and export are highly conserved between *C. elegans* and mammals. *C. elegans* express orthologs for DMT1 (SMF-3), ferritin (FTN-1, FTN-2), and ferroportin (FPN-1.1, FPN-1.2, FPN-1.3; **Figure 1**). The *C. elegans* genome also encodes potential orthologs for DCYTB ferrireductase and hephaestin multicopper oxidase. The intestinal anatomy in *C. elegans* is similar to vertebrates in that they contain an apical brush border facing the lumen and a basolateral membrane facing the interstitial space (circulation in mammals) (McGhee, 2013) (**Figure 1**). The intestine serves as the major site for absorption of dietary nutrients and a defense against xenobiotics and pathogens. *C. elegans* lack adipose tissue, liver, and pancreas and the intestine fulfills these functions by serving as a major site of lipid and glucose metabolism. Unlike mammals, *C. elegans* are heme auxotrophs and are dependent on acquiring heme from the environment (Rao et al., 2005; Hamza and Dailey, 2012; Yuan et al., 2013).

SMF-3 is the principal intestinal Fe(II) transporter in *C. elegans*. Consistent with its role in intestinal iron transport, SMF-3 is highly expressed at the apical membrane of intestinal epithelium (Au et al., 2009; Bandyopadhyay et al., 2009), transcriptionally activated during iron deficiency (Romney et al., 2011) and loss of SMF-3 expression leads to reduced iron content in *smf-3(ok1035)* null mutants (Romney et al., 2011). SMF-3 also transports Mn(II) as demonstrated by reduced Mn content in *smf-3(ok1035)* mutants (Romney et al., 2011), increased tolerance of* smf-3(ok1035)* mutants to Mn overload (Au et al., 2009) and Mn-mediated reduction in *smf-3* mRNA and SMF-3 protein in intestine (Au et al., 2009; Settivari et al., 2009). Like SMF-3, DMT1 transports Mn(II), which competes with Fe(II) uptake (Gunshin et al., 1997; Illing et al., 2012). The DMT1-deficient Belgrade rat displays impaired Mn uptake in intestine and erythroid precursors consistent with a physiological role for DMT1 in Mn uptake in mammals (Chua and Morgan, 1997). In excess, manganese is toxic, and in humans chronic occupational nasopulmonary exposure to Mn causes a neurological disease known as manganism (Roth and Garrick, 2003). Because Mn(II) and Fe(II) compete for DMT1 transport, this suggests that iron deficiency may be an important factor in the predisposition to Mn toxicity. Consistent with this are studies showing that iron deficiency is associated with increased Mn content in the brain of rats (Chua and Morgan, 1996; Erikson et al., 2002), in the olfactory epithelium of the DMT1-deficient Belgrade rat (Thompson et al., 2007) and in serum of humans with anemia or an iron deficient diet (Davis et al., 1992; Rahman et al., 2013).

*Caenorhabditis elegans* also express DMT1-like proteins SMF-1 and SMF-2 that share about 55–58% amino acid identity with DMT1 (Settivari et al., 2009). SMF-1 is widely expressed, but showed high expression in the apical intestinal membrane (Au et al., 2009; Bandyopadhyay et al., 2009), whereas SMF-2 is mainly cytoplasmic with high expression in pharyngeal epithelium (Au et al., 2009). *smf-3* and *smf-1* are transcriptionally induced upon exposure to pathogenic *Staphylococcus aureus,* and *smf-3(ok1035)*, and *smf-1(ok1748)* mutants showed hypersensitivity to this pathogen, indicating a role for these proteins in innate immunity (Bandyopadhyay et al., 2009). Like *smf-3*, exposure to high Mn reduces *smf-1* and *smf-2* mRNA levels, suggesting that reduced expression of these transporters may be a mechanism to reduce Mn toxicity (Settivari et al., 2009). This is consistent with a study showing that SMF-1 expression in dopamine neurons contributes to Mn^2^^+^-mediated neuronal death (Settivari et al., 2009). The roles of SMF-1 and SMF-2 in iron metabolism are not well understood; however, unlike *smf-3* mutant worms, iron and manganese content were not significantly reduced in *smf-1* and *smf-2* mutants compared to wildtype worms consistent with a prominent role of SMF-3 in iron and manganese transport (Romney et al., 2011).

The mechanism regulating basolateral transfer of iron to the interstitial space and to tissues in *C. elegans* is not known. In mammals, ferroportin is the sole exporter of iron to the circulation. *C. elegans* express three ferroportin orthologs, FPN1.1, FPN-1.2, and FPN-1.3, but their specific roles in iron export remains to be determined.

*Caenorhabditis elegans* express genes orthologous to human ferritin heavy subunit (*FTH*) and ferritin light subunit (*FTL*) genes. Ferritin is a ubiquitously expressed protein that stores iron in a form that is unable to generate free radicals. Mammalian ferritin is composed of a mixture of 24 FTL and FTH subunits that form a shell containing up to 4500 iron atoms (Theil, 2013). FTH exhibits ferroxidase activity that facilitates oxidation of iron, while FTL participates with FTH in the nucleation of iron (Bou-Abdallah, 2010; Liu and Theil, 2005). *C. elegans* FTN-1 and FTN-2 are more similar to human FTH than to FTL and both FTN-1 and FTN-2 contain ferroxidase active-site residues (Gourley et al., 2003). *ftn-1* is highly expressed in intestine whereas *ftn-2* is expressed in many tissues such as pharynx, body-wall muscle, hypodermis and intestine (Gourley et al., 2003; Kim et al., 2004). *ftn-1,* and to a lesser extent *ftn-2*, are induced by high iron exposure (Gourley et al., 2003; Kim et al., 2004). Only *ftn-1* mutants are iron sensitive and have reduced lifespans when exposed to high iron (Kim et al., 2004; Valentini et al., 2012).

Iron induces ferritin expression in mammals and in *C. elegans*, but the mechanism regulating ferritin differs in these organisms. In mammals, ferritin is primarily regulated at the translational level by iron-regulatory proteins 1 and 2 (IRP1 and IRP2) (Hentze et al., 2010; Anderson et al., 2012). During iron deficiency, IRPs bind to an RNA stem-loop known as the iron-responsive element (IRE) in the 5′ untranslated regions of *FTH* and *FTL* mRNAs to repress ferritin synthesis. When cellular iron increases, IRP1 is converted to its Fe-S cluster aconitase form concomitant with loss of RNA-binding activity, while IRP2 is targeted for ubiquitination and proteasomal degradation causing ferritin synthesis to increase (Salahudeen et al., 2009; Vashisht et al., 2009). *C. elegans* lack the IRP-IRE system, but express a cytosolic aconitase (ACO-1; Gourley et al., 2003; Kim et al., 2004). ACO-1 is homologous to mammalian IRP1 and its aconitase activity is regulated by iron, but unlike IRP1, it lacks RNA-binding ability. Despite lacking IRP-IRE regulation, *C. elegans* have evolved unique mechanisms to regulate iron storage.

## HIF-1 REGULATES IRON UPTAKE AND STORAGE DURING IRON DEFICIENCY

In *C. elegans*, hypoxia signaling is the predominant mechanism for regulating iron metabolism (Romney et al., 2011; Ackerman and Gems, 2012). Hypoxia signaling is a highly conserved process that conditions organisms to low oxygen and iron environments by regulating diverse biologic processes, including glucose metabolism, angiogenesis and iron metabolism (Semenza, 2007; Kaelin and Ratcliffe, 2008). During iron deficiency in mammals, hypoxia-inducible factor 2α (HIF-2α, also known as EPAS1) activates the transcription of *DMT1*, *FPN1* and *DCYTB* genes in the intestine to increase iron absorption (Taylor et al., 2011; Mastrogiannaki et al., 2009; Shah et al., 2009). Hypoxia-inducible factors (HIF-1 and HIF-2) are basic helix-loop-helix (bHLH) transcription factors that consist of oxygen-regulated α subunits (HIF-1α and HIF-2α) and a constitutively expressed β subunit (HIF-1β, also known as aryl hydrocarbon nuclear translocator or ARNT) (Semenza, 2007; Kaelin and Ratcliffe, 2008; Kaluz et al., 2008). Under normal conditions, in the presence of oxygen and iron, HIF-α subunits are hydroxylated by prolyl hydroxylase (PHD2, also known as EGLN1) whose activity is dependent upon oxygen and iron. Hydroxylated HIF-α is targeted for proteasomal degradation by the E3 ubiquitin ligase von Hippel Lindau tumor suppressor protein (VHL) (Ivan et al., 2001). During hypoxia or iron deficiency, PHDs are inactive, thus allowing HIF-α subunits to translocate to the nucleus, dimerize with HIF-1β and recruit coactivators to activate target gene expression in pathways such as erythropoiesis, iron metabolism, glucose metabolism and angiogenesis (Semenza, 2007; Kaelin and Ratcliffe, 2008; Kaluz et al., 2008). HIF-1α and HIF-2α regulate overlapping, but distinct sets of target genes (Kaluz et al., 2008). For example, only HIF-2α is responsible for the coordinate upregulation of *DMT1*, *DCYTB* and *FPN1* in intestine during iron deficiency (Mastrogiannaki et al., 2009; Shah et al., 2009; Taylor et al., 2011). HIF-2α regulation of intestinal iron metabolism during iron deficiency ensures that sufficient iron is absorbed and delivered to the bone marrow for production of red blood cells (Shah and Xie, 2014).

The HIF signaling pathway is conserved in *C. elegans*. *C. elegans* express HIF-1, AHA-1, VHL-1, and EGL-9, which are orthologs of HIF-1α/HIF-2α, HIF-1β, VHL and PHD, respectively, in vertebrates (Epstein et al., 2001; Jiang et al., 2001). Unlike mammals, *C. elegans* express a single *hif-1* gene that shares homology to *HIF1α* and *HIF2α* (Jiang et al., 2001). HIF-1 functions in a variety of biological processes ranging from stress response, innate immunity, neuronal development, ageing and iron metabolism as discussed below (Shen et al., 2005; Chang and Bargmann, 2008; Pocock and Hobert, 2008; Luhachack et al., 2012; Jones et al., 2013).

**FIGURE 2:**
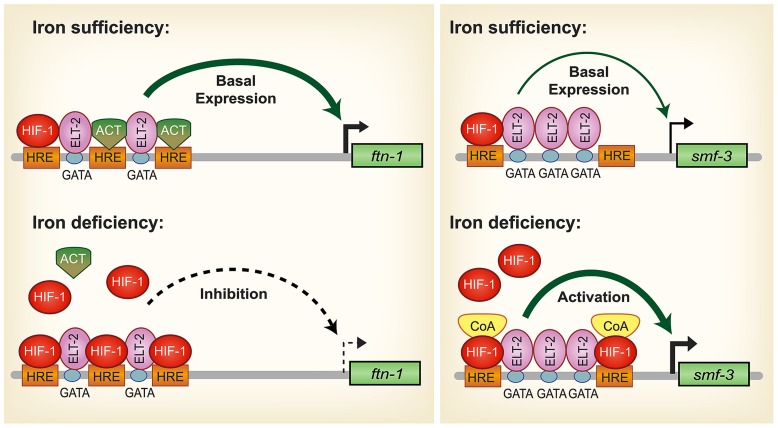
**Model for HIF-1 iron-dependent activation and inhibition of intestinal iron uptake and storage in *C. elegans*.** *Iron sufficiency*: ELT-2 binds to GATA binding sites located in the *ftn-1* and *ftn-2* IDEs. We propose that ELT-2 cooperates with an unidentified transcriptional activator (ACT) that binds to the hypoxia-response elements (HREs) to regulate transcription. As HREs resemble E-box elements, which are binding sites for bHLH transcription factors, it is possible that a bHLH transcription factor serves this role. *smf-3* is transcribed at basal levels during iron sufficiency to limit iron absorption. HIF-1 is expressed during normal growth conditions, but at low levels. *Iron deficiency*: HIF-1 accumulates and dimerizes with AHA-1. HIF-1/AHA-1 (denoted by HIF-1) displaces the transcriptional activator ACT binding to the *ftn-1* and *ftn-2* HREs and inhibits transcription. Another possible mechanism for HIF-1 mediated *ftn-1* repression is the displacement of ELT-2 by HIF-1. HIF-1/AHA-1 binds to the *smf-3* HREs, recruits coactivators (CoA) and cooperates with ELT-2 to activate *smf-3* transcription. Whether ELT-2 is bound to the *ftn-1* GATA sites during iron deficiency and to the *smf-3* GATA sites during iron sufficiency has not been determined. (Adapted from Romney et al., 2011).

During iron deficiency, *ftn-1* and *ftn-2* transcription is repressed and is dependent upon a *cis*-regulatory element termed the iron-dependent enhancer (IDE) located in the *ftn-1* and *ftn-2* promoters (Kim et al., 2004; Romney et al., 2008) (**Figure 2**). Basal expression of *ftn-1* and *ftn-2* is mediated by the intestinal GATA transcription factor ELT-2 that binds GATA sites located in ferritin IDEs (Romney et al., 2008). Further studies revealed that HIF-1 binds to hypoxia response elements (HREs) located in the IDEs of *ftn-1* and *ftn-2* to repress transcription during iron deficiency (Romney et al., 2011; Ackerman and Gems, 2012). Intestinal iron uptake through SMF-3 is also regulated by HIF-1 during iron deficiency. Similar to *ftn-1* and *ftn-2* IDEs, *smf-3* contains an IDE in its promoter that contains HRE binding sites that confer HIF-1 dependent activation during iron deficiency (Romney et al., 2011) (**Figure 2**). Romney et al. (2011) also showed that *hif-1 (ia04)* mutants have reduced iron and manganese content and are developmentally delayed when grown in iron deficient conditions. Notably, development of *hif-1(ia04)* mutants was restored when the cellular iron pool was increased by RNAi depletion of *ftn-1* and *ftn-2.* It is not known whether the ferroportin homologs *fpn-1.1, fpn-1.2* and *fpn-1.3* and *DCYTB* homologs are regulated by hypoxia. These studies show that regulation of iron uptake and storage by HIF-1 is crucial for ensuring proper growth and development during iron deficiency.

HIF-1 is well known as a transcriptional activator but less is known about its role as a transcriptional repressor. The question arises regarding the mechanism of HIF-1 transcriptional repression of *ftn-1* and *ftn-2.* Chromatin immunoprecipitation analysis and electrophoretic mobility gel assays showed direct HIF-1 binding to the *ftn-1* IDE (Romney et al., 2011; Ackerman and Gems, 2012). Another study showed that mutations of all three HREs in the *ftn-1* IDE abolished expression of a *pftn-1::gfp* transcriptional reporter, suggesting that an activator may bind the HREs during normal conditions (Romney et al., 2011; **Figure 2**). HREs resemble E-box elements and it is possible that this activator may be a member of the basic helix loop helix (bHLH) transcription factor family that can bind to non-canonical E-boxes (Kewley et al., 2004). A MAD-like transcription factor MDL-1 was identified in an RNAi screen as a transcriptional activator of *ftn-1* expression (Ackerman and Gems, 2012). *mdl-1* encodes a bHLH transcription factor that bind E-box sequences as a dimer with MXL-1 to regulate target genes(Yuan et al., 1998). MDL-1 transcriptional regulation of *ftn-1* was shown to be iron independent (Ackerman and Gems, 2012), suggesting the possibility that MDL-1 may bind to the *ftn*-1 and *ftn-2* HREs when iron is sufficient, but is displaced by HIF-1 when iron is low. Alternatively, it is possible that during iron deficiency the displacement of ELT-2 from its GATA binding sites by HIF-1 results in decreased *ftn-1* and *ftn-2* transcription. Further work is required to define this mechanism. In mammals, ferritin has not been reported to be regulated by HIF-2α; however, hypoxia regulates ferritin expression by altering IRP1 RNA binding activity and IRP2 protein abundance (Schneider and Leibold, 2003; Meyron-Holtz et al., 2004; Salahudeen et al., 2009; Vashisht et al., 2009).

## FERRITIN REGULATION BY THE INSULIN/INSULIN-LIKE GROWTH FACTOR SIGNALING PATHWAY

**FIGURE 3:**
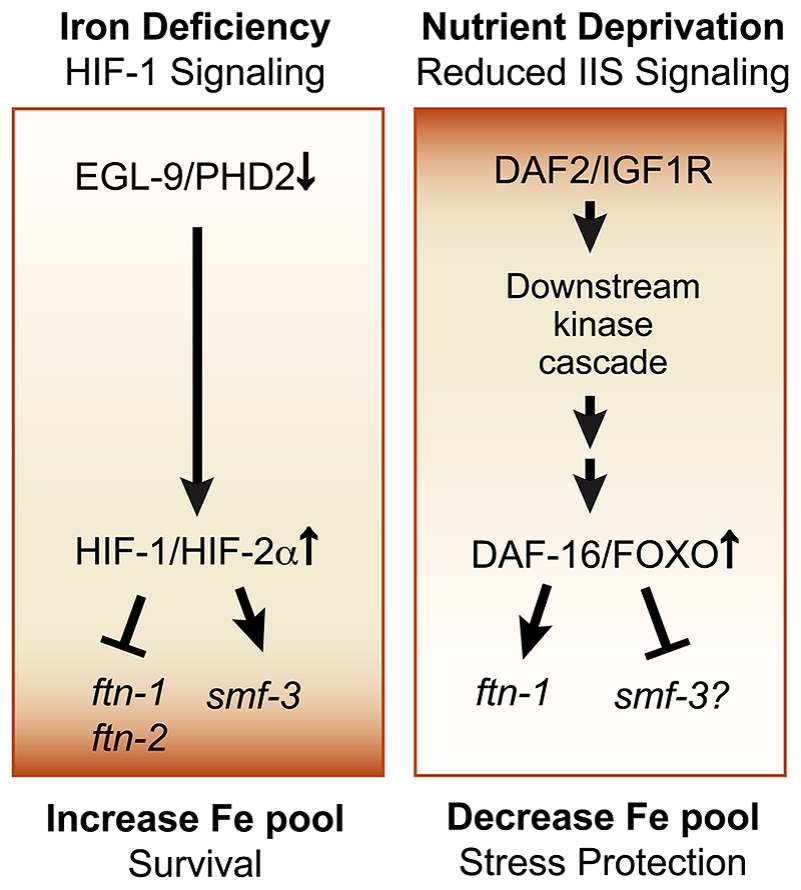
**Pathways regulating cellular iron metabolism in *C. elegans*.** *Iron deficiency-HIF-1 pathway:* this pathway is activated in response to iron deficiency or hypoxia and results in HIF-1 dependent repression of *ftn-1* and *ftn-2* and activation of *smf-3*. This leads to reduced iron storage and increased iron uptake, ultimately increasing the cellular iron pool and promoting survival during iron limitation. *Insulin/insulin growth factor-1 (IGF-1) signaling (IIS) pathway:* the IIS pathway is a conserved pathway in worms, flies and in vertebrates that regulates the transcription factor DAF-16/FOXO. DAF-16/FOXO regulates genes in essential processes, such as metabolism, stress resistance, pathogen defense and lifespan extension. Activated IIS initiates a phosphorylation cascade that leads to the cytoplasmic retention of DAF-16/FOXO and inhibits its function. Nutrient deprivation reduces IIS leading to the nuclear localization of DAF-16 to activate *ftn-1.* Glucose upregulates *smf-3* expression, but whether DAF-16 directly regulates *smf-3* remains to be determined. This pathway provides a mechanism to increase cellular iron by reducing *ftn-1* when nutrients are abundant to promote growth and to reduce cellular iron during stress by increasing *ftn-1* to limit iron-catalyzed oxidative stress.

Ferritin is regulated by the insulin/insulin-like (IIS) growth factor signaling pathway in *C. elegans*. The IIS pathway is a conserved pathway in vertebrates and *C. elegans* that coordinates nutrient availability with development, metabolism and stress responses (Accili and Arden, 2004; **Figure 3**). When nutrients are available, insulin and insulin-like growth (IGF) factors activate tyrosine kinase receptors DAF-2/IGFR1, triggering a kinase cascade that leads to the phosphorylation of the Forkhead box, Class O (FOXO) transcription factor DAF-16/FOXO and its cytoplasmic retention and inhibition. When IIS is reduced during nutrient deprivation, DAF-16/FOXO phosphorylation is reduced, promoting DAF-16/FOXO translocation to the nucleus where it regulates the expression of target genes involved in stress resistance, metabolism, and innate immunity (Murphy and Hu, 2013). A recent study showed that *ftn-1* expression was elevated in *daf-2* mutants compared to *daf-16;daf-2* mutants, indicating that DAF-16 activated *ftn-1* expression (Ackerman and Gems, 2012). Further genetic studies showed that *hif-1* and *daf-16* act in parallel pathways to regulate *ftn-1* and that DAF-16 regulation of *ftn-1* was not iron dependent (Ackerman and Gems, 2012). Less is known about the role of IIS in *smf-3* regulation. One study showed that glucose treatment induced the *smf-3* expression, suggesting a potential role for IIS and DAF-16 in *smf-3* downregulation (Lee et al., 2009). Reduced IIS leads to DAF-16 dependent upregulation and downregulation of a diverse set of genes, which are designated as class 1 and class II genes, respectively (Lee et al., 2003; McElwee et al., 2003; Oh et al., 2006). More recently, the transcription factor PQM-1 was discovered to regulate class II genes by binding to the DAF-16 associated element (DAE) located in the promoter of these genes, whereas DAF-16 regulates class 1 genes by binding to the DAF-16 binding element (DBE; Tepper et al., 2013). The *smf-3* promoter contains both DBE and DAE binding sites, but whether DAF-16 or PQM-1 regulates *smf-3* awaits future studies. Taken together, these studies suggest that DAF-16 activation of *ftn-1* during reduced IIS provides *C. elegans* with a mechanism to increase iron storage, thereby limiting iron toxicity during stress conditions (**Figure 3**). When IIS is stimulated, DAF-16 is inhibited and *ftn-1* transcription is reduced, increasing the availability of iron required for development and growth

Insulin signaling and FOXO regulation of mammalian ferritin has not been reported. However, mammalian ferritin is transcriptionally activated by oxidative stress (Thimmulappa et al., 2002; Pietsch et al., 2003a,b; Hintze and Theil, 2005) and repressed by oncogenes, providing a mechanism to sequester iron during stress and to increase iron availability during cell proliferation (Tsuji et al., 1993, 1995; Wu et al., 1999). Similarly, several studies have shown that ferritin depletion stimulates cell proliferation by increasing available iron, whereas sequestration of iron by ferritin overexpression slows cell proliferation (Cozzi et al., 2000; Kakhlon et al., 2001; Cozzi et al., 2004; Baldi et al., 2005). Like *C. elegans*, changes in ferritin expression in response to environmental stimuli are essential for survival during stress and growth during normal conditions.

## OTHER REGULATORS OF FERRITIN EXPRESSION

*ftn-1* transcription has also been shown to be repressed by the REF-1-like protein HLH-29 (Quach et al., 2013) and UNC-62 a member of the TALE family of homeobox transcription factors (Catoire et al., 2011; Ackerman and Gems, 2012). HLH-29 is homologous to the HairyVEnhancer of Split (HES) transcription factors that regulate embryonic development through Notch-dependent and independent pathway (Fischer and Gessler, 2007). HLH-29 was recently shown to bind promoter sequences upstream of the *ftn-1* IDE and repress its transcriptional expression independent of the iron responsive HIF pathway (Quach et al., 2013). Additionally, *hlh-29* mutants have elevated levels of *ftn-1* and are resistant to peroxide stress. Further studies are needed to define the mechanism and significance of this regulation.

*unc-62* encodes the mammalian ortholog of MEIS1 that has a crucial role in normal development and in leukemia (Azcoitia et al., 2005; Argiropoulos et al., 2007). MEIS1 has also been identified as a Restless Leg Syndrome (RLS) predisposing gene (Winkelmann et al., 2007; Xiong et al., 2009; Spieler et al., 2014). RLS is a sensorimotor disorder that is associated with iron insufficiency in brain, but the role of iron in RLS is not well understood (Clardy et al., 2006; Allen and Earley, 2007; Catoire et al., 2011). It is of interest that *ftn-1* expression is significantly decreased in *C. elegans* treated with *unc-62* RNAi (Ackerman and Gems, 2012), suggesting that dysregulation of MEIS-1/MEIS can lead to altered iron metabolism.

Ferritin regulation spans beyond iron and nutrient stress. For instance, *ftn-2*, but not *ftn-1*, was shown to be necessary for proper innate immune response to pathogenic *S. aureus* (Simonsen et al., 2011). During infection, *ftn-2* was also transcriptionally upregulated along with several DAF-16 targets. It is likely that DAF-16 activates *ftn-2* to protect *C. elegans* from bacterial infection by limiting iron availability.

## CONCLUDING REMARKS

We have highlighted recent studies showing the potential of *C. elegans* as a useful genetic platform to explore mechanisms integrating iron and oxygen metabolism. Future genomic studies are needed to identify additional target genes of HIF-1 that are specific to hypoxia or iron deficiency and the unique HIF-1 partner proteins that coordinate these responses. A better understanding of how iron and insulin signaling are coordinated in *C. elegans* could provide new knowledge about the role of iron in glucose metabolism and in the pathogenesis of diabetes in humans. Finally, these studies have set a foundation for the development of genetic screens to identify novel regulators that are involved in iron sensing, uptake, storage and utilization. *C. elegans* holds promise as a system to decipher complex pathways regulating iron metabolism that can be followed up in mammals.

## Conflict of Interest Statement

The authors declare that the research was conducted in the absence of any commercial or financial relationships that could be construed as a potential conflict of interest.
